# Our incision types of choice for surgical treatment of true aneurysms of subclavian and axillary arteries

**DOI:** 10.1186/1749-8090-8-S1-P49

**Published:** 2013-09-11

**Authors:** O Gokalp, I Yurekli, L Yilik, U Yetkin, T Gunes, M Akyuz, B Ozcem, O Tetik, G Ilhan, A Gurbuz

**Affiliations:** 1 Department of Cardiovascular Surgery, Izmir Katip Celebi University Ataturk Training and Research Hospital, Izmir, Turkey

## Background

Subclavian and axillary arteries true aneurysms are rarely seen. The best incision for surgical approach is median sternotomy for intrathoracic right subclavian artery aneurysm, left thoracotomy for intrathoracic left subclavian artery aneurysm and supraclavicular incision for extrathoracic ones. Many surgeons approaches as subclavicular incision, subclavicular incision extending to axillary incision, anterolateral incision and posterolateral incision are also commonly used.

## Methods

Eight patients that were operated on between February 1998 and December 2007 due to true aneurysms of the subclavian and axillary arteries were examined. Two of these patients had subclavian and 6 of them had axillary artery aneurysms.

## Results

We used subclavicular incision in one and supraclavicular incision in the other patient for operating the subclavian artery aneurysm. But median sternotomy was added to the supraclavicular incision in the second operation of the patient who developed pseudoaneurysm after the first operation with a single supraclavicular incision. In axillary artery aneurysms, however, infraclavicular, deltoidopectoral and subpectoral incisions are generally used. In our study, subclavicular approach was used.

## Conclusions

Subclavian and axillary artery aneurysms should be promptly treated surgically after diagnosis. The localization of the aneurysm is the primary determinant of the incision.

